# Attitudes towards seeking psychological help among community dwelling older adults enrolled in primary care in Chile

**DOI:** 10.1186/s12877-024-04986-3

**Published:** 2024-05-01

**Authors:** Ximena Moreno, Francisco Moreno

**Affiliations:** 1 Facultad de Psicología y Humanidades, Universidad San Sebastián, Lota 2465 Providencia, Santiago, Chile; 2 Department of Mathematics and Computer Science, University of Santiago, Las Sophoras 175, Oficina 420, Estación Central, Santiago, Chile

**Keywords:** Psychological help, Attitudes, Common mental disorders, Primary care, Help-seeking

## Abstract

**Background:**

Depression and anxiety are common mental disorders among older adults, but they are frequently underdiagnosed. Attitudes towards seeking professional mental health care is one of the barriers to access to treatment. This study was aimed at assessing the attitudes towards seeking psychological help among older adults who are enrolled in primary care in Chile, and to determine the associated factors.

**Methods:**

This cross-sectional study recruited 233 primary care users aged 65 or more years. The Attitudes Towards Seeking Professional Psychological Help was used. Reliability and factor analysis of this scale were carried out. The average scores of the scale and factors were calculated and compared, by selected variables. Multivariate linear regression was estimated to determine factors associated with attitudes towards seeking psychological help.

**Results:**

Three factors were identified in the attitudes towards seeking psychological help: confidence in psychologists, coping alone with emotional problems, and predisposition to seek psychological help. On average, participants had a favorable attitude towards seeking psychological help, compared with previous research. Lower level of education, and risk of social isolation were inversely associated with these attitudes.

**Conclusion:**

Strategies to improve mental health literacy and social connection among older adults, could have an impact on factors that mediate the access to mental health care, such as attitudes towards seeking psychological help, among people who have a lower level of education or are at risk of social isolation.

**Supplementary Information:**

The online version contains supplementary material available at 10.1186/s12877-024-04986-3.

## Introduction

Depression and anxiety are the two most common mental disorders, and the leading mental health causes of years lost to disability (YLD), and disability adjusted life years (DALY) among older adults [1]. Considering all causes of YLD globally, depressive disorders are the fourth and the fifth cause of YLD among women and men aged 50 to 69 years, respectively [1]. These disorders have an impact on quality of life in old age [2], and they increase the risk of adverse health outcomes, including incident disability and mortality [3–5].

Common mental disorders are frequent among older adults who are primary care users [6], but they are underdiagnosed [7, 8]. Older people experience barriers to access mental health care, which are associated with multiple factors such as the workload in primary care and scarce time available to build a relationship with users, or reduced access and communication with specialty mental health services, which decreases the likelihood of referral [9]. Lack of training and experience among health providers may interfere with the recognition of these conditions in older adults [10], since specific knowledge and communication skills are needed to obtain and interpret relevant information for diagnosis [11]. Some symptoms of depression and anxiety in old age may be confounded with the normal features of the aging process, or with expressions of physical health problems that cause distress [7, 12]. Also, as reported by a systematic review, stereotypical negative views about old age among health providers may result in direct discrimination, including the lower likelihood to refer older adults to psychological therapies [13]. Previous studies have found that mental health professionals considered that older adults were less likely to benefit from psychotherapy than younger people with similar symptoms [14].

The existing evidence has shown that psychotherapy can be effective to treat depression among older adults [15–17]. Although Cuijpers et al. [16] findings did not support a greater efficacy of psychotherapy for depression among older adults, compared to younger adults, a recent study in England found that psychotherapy was more effective to reach recovery from depression and anxiety among older adults, compared to working age adults [18]. This is of particular interest for older adults who prefer non-pharmacological treatments and those who are more vulnerable to negative side effects due to multibormodibity, frailty and interaction with other medications in case of polypharmacy [19]. Preference for psychotherapy versus pharmacological treatment for mental health conditions has been reported more often among younger age groups [20, 21], but previous research suggests that older adults who live in the community tend to prefer counseling or psychotherapy [22, 23]. Nevertheless, a study in Germany found that among older adults with a perceived need for mental health treatment, more than half did not utilize psychotherapy [24]. A review about barriers for older adults to seek professional help for mental health problems, reported stigma about mental health problems, negative beliefs about the efficacy of treatments, and cost, as the main factors associated with a lower likelihood of seeking professional help [25].

Most studies about the attitudes towards seeking professional psychological help among older adults have been carried out in the United States and Europe [25, 26]. In Latin America, a Mexican study found that males, people who were not health care users and those who believed that depression was not a disease were less likely to search mental health care [27]. To date, there are no studies in Chile that have assessed the attitudes towards seeking professional mental health care among older adults. Previous Chilean studies have analyzed the psychometric properties of scales to measure these attitudes, but they have focused on adolescents and university students [28, 29].

According to studies based on population data, the prevalence of positive screen for depressive symptoms in older adults in Chile reached 28.3% (23.2% of men; 31.6% of women) in 2009 [30], and 29.2% (20.9% of men; 36.4% of women) in 2016 [31]. In Chile, 84.9% of older adults are primary care users [32], where they undergo the Annual Preventive Medical Evaluation of the Elderly, aimed at identifying people at risk of dependency or dependent, among those aged 65 or more years [33]. Among several dimensions, this exam assesses anxiety and depression symptoms, to refer older adults to a physician for diagnosis confirmation [34]. However, the national coverage of this program reaches less than half of older adults enrolled in primary care [35], and a regional study reported a frequency below 15% of medical consultations to confirm the diagnosis of depression screened through this program [36]. Consistently, a study found that among older adults with a positive screen for major depressive disorder in Chile, less than half had received a diagnosis [31].

Since 2000, a community approach is central in mental health policies in Chile [37]. Primary care is the entry point and the main provider of mental health care, as part of a local network including secondary care facilities [37]. In this context, the National Program for Screening, Diagnosis and Comprehensive Treatment of Depression was implemented in all primary care centers in Chile in 2005 [38]. In 2005, depression in people aged 15 or more years was included in the Regime of Explicit Health Guarantees, a program aimed to improve the equity in the access, opportunity, quality, and financial protection for prioritized health conditions [39]. There is a protocol to treat depression in public health services, which includes consultations at different levels of the health system, depending on the severity of the disorder [38]. Also, people diagnosed in both the public and private systems can access the Regime of Explicit Health Guarantees program, which may include medical and psychological consultation, psychiatric consultation, medication, psychosocial interventions, and laboratory exams, according to the protocol [40]. As it occurred globally [41], the COVID-19 pandemic had an impact on the provision of mental health services in Chile. An analysis between 2019 and 2021 showed that mental health consultations in primary care had a decrease of 88% in 2020, compared to pre-pandemic levels, whereas secondary and tertiary mental health provision decreased by 66.3 and 71.3%, respectively [42]. With respect to psychological interventions, 60% of national health services reported a partial reduction, and in 40% of health services in the country the reduction was substantial [42]. Another study that analyzed admissions to mental health programs in 10 primary care centers from a municipality in Chile found that mental consultations decreased among people aged 50 or more years, comparing the year previous to the pandemic to 2020 [43]. Furthermore, a longitudinal analysis that compared positive screen for depression and anxiety among older adults in Chile, before and during the pandemic, reported an increase of both types of symptoms [44].

In Chile, psychologists have been part of the mental health team in primary care for more than two decades [45]. Nearly 80% of patients with mental health disorders are treated in primary care, where they usually receive individual treatment or group interventions from psychologists [37]. However, it is unknown how likely are older primary care users in Chile to seek psychological help. The aims of this study were to assess the attitudes towards seeking psychological help among older adults who are primary care users in Chile, and to determine the association between these attitudes and sociodemographic characteristics, health situation, depressive symptoms, and social support.

## Methods

### Participants of the study

The sample of the study (*n* = 233) was recruited among community dwelling older adults enrolled in primary care centers in the administrative area of the North Metropolitan Health Service, in Santiago, Chile. Potential participants were contacted from existing programs in the primary care centers, such as a program to prevent disability among older adults, or community centers for older adults. The inclusion criteria were having 65 years of age or more, and being enrolled in the primary care centers considered in the study. People with severe disabilities, bedridden or unable to answer an interview were excluded.

### Procedures

Potential participants were invited to a community center near their home, where they received detailed information about the study and their participation, prior to obtaining their signed informed consent. Additionally, people who could not attend the place of interview, were offered a home visit. Trained interviewers, with a social science or health professional degree, carried out face to face structured interviews, between July and December 2023. The interview length was 40 minutes, in average. A questionnaire was employed, including questions about sociodemographic characteristics, social support, health status, depression symptoms, and attitudes towards seeking psychological help.

### Measurements

#### Attitudes towards seeking psychological help

The Attitudes Toward Seeking Professional Psychological Help Scale-Short Form (ATSPPH-SF) was used, which is a 10-item version adapted from the original 29-item scale [46]. The response format consists of a four-point Likert-type scale from 0 to 3 (disagree, partly disagree, partly agree, and agree), and the total score ranges from 0 to 30, with higher scores indicating a more favorable attitude towards seeking psychological help. Although Fischer and Farina [46] found evidence of a one-factor structure among a college students sample, the short version of the scale included items from two of the dimensions of the original scale: “recognition of the need for psychological help”, and “confidence in mental health practitioner”, which theoretically supports the existence of two constructs [47]. Subsequent studies have assessed the psychometric properties of the ATSPPH-SF, but the results have shown inconsistency with respect to its factor structure, depending on time, sample, and cultural contexts. A study from Singapore, carried out with a sample of teachers in 2007 found that the item 7 of the scale, which is double-barreled, affected the goodness of fit of the one-factor model, and supported a nine-item one factor model [48]. A one-factor structure was also supported by a study carried out among university students in Jordania in 2016 and 2017 [49]. A study that recruited a sample of college students in 2005, and primary care users between 2005 and 2006 in the United States [47] reported a two-factor structure, with one factor referring to “openness to seeking treatment for emotional problems”, and another about “value and need in seeking treatment”. In China, a study with college students retained only seven items that had loadings above 0.30, considering these two factors, but the internal consistency of the seven-item scale was poor [50]. A more recent population based study in China [51] confirmed the two-factor structure found in the United States46 among college students and primary care users [47]. Torres et al. [52] reported a good fit of the two-factor structure, and supported the recommendation of removing the item 7. A study that analyzed a sample of the population aged 18–65 in Singapore, between 2014 and 2015, found that a three-factor structure had a better fit, including “openness to seeking professional help”, “value in seeking professional help”, and “preference to cope on one’s own” [53]. Finally, a recent study that a analyzed a convenience sample aged 10–65 years in Iran [54], found a two-factor structure similar to the findings of the study carried out in the United States in 2005 [47].

Since the ATSPPH-SF has not been used previously in Chile and among older adults, the original scale was translated from English into Spanish by a professional translator and by two psychologists with research experience. These versions were revised and harmonized by the researchers, and then back translated by native English speakers, who are fluent Spanish speakers. The researchers reviewed and compared the original translations and back translations, and discussed the differences with the translators, to develop the Spanish version of the scale. To improve language adequacy, the items in Spanish were read and discussed with four older adults, and they made comments about their wording and meaning. Considering these comments, and since the scale was going to be used in an interview, instead of self-applied, the subject pronouns were changed from singular first person (I) to singular second person (you) in items 1, 2, 3, 5, 6, and 8.

**Fig. 1:**
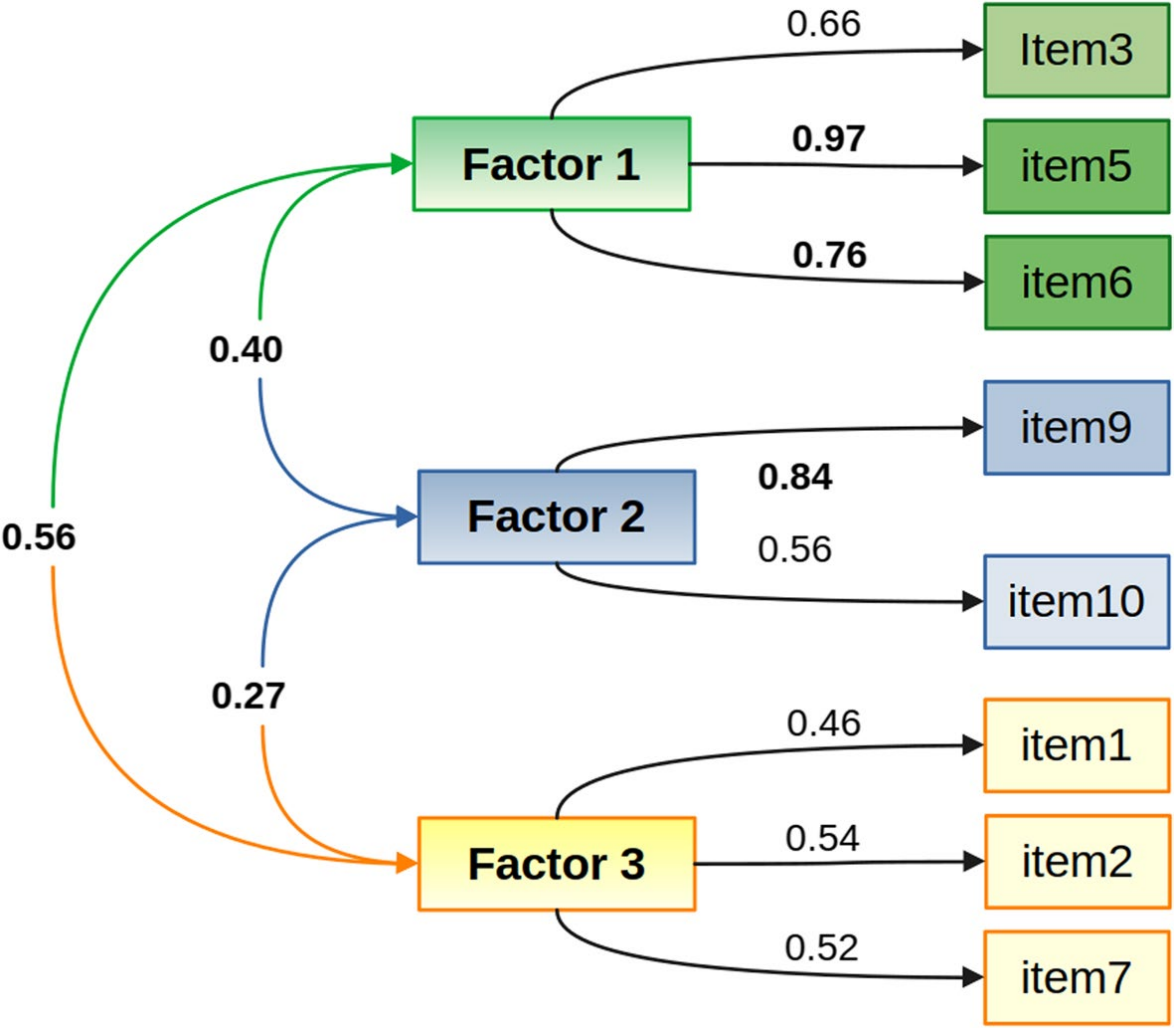
Three-factor model of the ATSPPH-SF

We used psych [55], and lavaan [56], packages in R [57] to assess the factor structure of this scale. According to Bartlett’s test of sphericity (*p* < 0.001), and Kaiser-Meyer-Olkin test (MSA = 0.80), the data were adequate to perform exploratory factor analysis (EFA). As recommended for ordinal data, polychoric correlations were calculated [58]. Minimal residual was used to estimate the factors [59]. We used an oblique rotation [60], which is suitable for psychological scales with correlated factors [59]. The EFA showed that the item 4, which refers to how admirable are people who try to solve their emotional problems on their own, without seeking professional help, and the item 8, that expresses that the value of psychotherapy is doubtful, considering how expensive and time consuming it is, did not have loadings above 0.3. Hence, these two items were removed, and after confirming the suitability of the data for EFA (*p* < 0.001 in sphericity test; MSA = 0.79), we found that a three-factor model explained 62% of the variability, had loadings> 0.45 for all items, and showed no cross-loadings on factors (Fig. 1). Confirmatory factor analysis (CFA), comparing the fit indices of one, two, three, and four factor models, showed a better fit for the three-factor model (*X*^*2*^ (22, *N* = 233) = 35.2, *p* = 0.037; CFI = 0.973; TLI = 0.966; RMSEA = 0.051; SRMR = 0.078). According to this model, the first factor referred to the confidence in psychologists and psychological help, and included items 3, 5, and 6. Items 9 and 10, with higher loads on the second factor, expressed beliefs about the need of coping alone or not with emotional problems. The third factor included items 1, 2, and 7. In this case, the factor was associated with the predisposition to seek psychological help, with item 1 expressing the immediate tendency, in case of experiencing emotional problems, and items 2 and 7, referring to the value of receiving psychological help. Previous research has reported Cronbach’s alpha as a measure of reliability of the ATSPPH-SF [50–52]. In our case, the alpha of the 8-item scale was 0.77, and considering the items of each factor, the alpha was 0.82 (factor 1), 0.57 (factor 2), and 0.52 (factor 3). Nevertheless, in spite of being frequently reported, alpha involves a unidimensional analysis that assumes an uncorrelated factor structure, which is not met in psychological scales [61]. McDonald’s omega statistic, in turn, is based on pairwise item covariances, which allows to attribute omegas associated with factors as an estimation of the reliability of each factor [62]. Due to the limitations of Cronbach’s alpha, McDonald’s omega is among the recommended measures of reliability for multidimensional scales [63]. Hence, we assessed the reliability of the 8-item scale with McDonald’s omega, which was 0.90. The coefficient omega for each factor was: 0.90 (factor 1), 0.71 (factor 2), and 0.69 (factor 3). Although a cutoff for good reliability from omega has not been described in the literature, it is possible to observe that the internal consistency of factors 2 and 3 was lower. The 8-item scale had a total score ranging from 0 to 24. The original 10-item scale was used for sensitivity analysis.

### Sociodemographic characteristics

In this set of variables, we included:

- Age, categorized as less than 75, and 75 or more years.
- Gender (male or female).
- Level of education, which was categorized as incomplete primary education, complete primary education, and complete secondary education or more.
- Marital status, categorized as single, married or with a partner, divorced, and widowed.
- Living alone or not.
- Perceived economic difficulties during the last month, where people were asked to report if, during the last month, the total household income was a) enough, and they experienced no difficulties, b) enough, without important difficulties, c) not enough, they had some difficulties, d) not enough, they had many difficulties. The answers were categorized as “no economic difficulties” (a + b), and “economic difficulties” (c + d).

### Health situation

- Self-rated health was assessed with the question: “In general, would you say that your health is? The response categories were “very good”, “good”, “fair”, “poor”, “very poor”. The answers were collapsed into “good” and “less than good”, considering fair or poor.
- Activity limitation, measured with the Spanish version of the global activity limitation indicator [64]: “For at least the past six months, have you had to limit the activities you usually do, because of a health problem? The answer categories were “yes, very much”, “yes, a bit” or “no, I have not”. They were categorized as “yes”, including the first two categories, or “no”.
- Primary care visit regularity, considering the categories: “once a month or more”, “less than once a month”.
- To determine the knowledge about the mental health program in primary care, people were asked if they had information about the type of mental health problems included in the primary care programs. The possible answers were “yes” or “no”.

### Depressive symptoms

To assess mental health status, we employed the 9-item Patient Health Questionnaire, which has been validated among older adults who are primary care users in Chile [65]. The total score ranges from 0 to 27, and a cutoff of 10 or more is recommended as a positive screen for major depressive disorder [66]. Hence, two categories were considered: “negative screen for depression” (0–9 points), and “positive screen for depression” (10 or more points). We also carried out sensitivity analyses with a cutoff of 6 or more points, as suggested by previous studies considering samples of older adults [65, 67].

### Social support

The 6-item Lubben Social Network Scale (LSNS-6) was used to measure social support. The total score of the scale ranges from 0 to 30, and a score of 12 or less indicates risk of isolation [68].

### Analysis

The distribution of the main characteristics of the sample was described. The mean and standard deviation of the total score of the ATSPPH-SF, and the three factors identified, were calculated, Group comparisons were carried out with t-test or ANOVA, depending on the number of categories of each variable.

To determine the factors associated with attitudes towards seeking psychological health, regression models were estimated, considering the 8-item scale. A model with the original 10-item scale as the outcome was calculated in sensitivity analysis. Multicollinearity was assessed with variance inflation factor (VIF). The mean VIF was 1.3, with values ranging from 1.06 to 1.67.

These analyses were performed with Stata 18.0 and R.

### Ethical considerations

The protocol of this study was approved by the Ethics Committee of the North Metropolitan Health Service. All participants signed an informed consent before taking part in the study.

## Results

As observed in Table 1, the sample included almost three thirds of women, and 60% of people aged less than 75 years. Participants more frequently have completed secondary education (62,23%), but 13.73% of them have less than primary education. The most frequent marital status was “married or with a partner” (40.77%), followed by widowhood (28.33%). One fourth of the people interviewed lived alone, and 36.05% reported economic difficulties during the last month. With respect to health status, 42.06% reported their health was less than good, and 48.07% had limitations in their daily activities. People who visited at least once a month the primary care center reached 60.52%, but slightly more than a quarter declared knowledge about the mental health program (27.95%). Depressive symptoms were detected in 15.45%. More than half of the people interviewed were at risk of social isolation (52.36%).

**Table 1:** Characteristics of the sample

Variable	n (233)	%
Gender		
Male	62	26.61
Female	171	73.39
Age		
Below 75	141	60.52
75 and above	92	39.48
Education		
Less than 8 years	32	13.73
Complete primary education	56	24.03
Complete secondary education or more	145	62.23
Marital status		
Married or with partner	95	40.77
Single	31	13.30
Divorced	41	17.60
Widowed	66	28.33
Living alone		
Yes	59	25.32
No	174	74.68
Economic difficulties		
Yes	84	36.05
No	149	63.95
Self-rated health		
Good	135	57.94
Less than good	98	42.06
Activity limitation		
Yes	112	48.07
No	121	51.93
Frequency of visits to primary care		
Monthly	141	60.52
Less than once a month	92	39.48
Knowledge about mental health program		
Yes	64	27.95
No	165	72.05
Depressive symptoms		
Yes	36	15.45
No	197	84.55
Social support		
No risk of isolation	111	47.64
At risk of isolation	122	52.36

The mean score of the 8-item ATSPPH-SF was 19.28 (SD 3.97). According to the sensitivity analysis with the 10-item original scale (See Supplementary Table 1, Additional file 1), the mean was 21.84 (SD 4.67). Factor 1, that referred to confidence in psychologists and psychological help, had an average score of 7.23 (SD 2.11). Factor 2, expressing beliefs about the importance of not coping alone with emotional problems, had a mean of 4.22 (SD 1.60). The average score for Factor 3, about the predisposition to seek for psychological help, was 7.82 (SD 1.47). The scores for each subgroup are depicted in Table 2. People with activity limitation had a higher mean score (19.81), compared to those without limitation (18.79). With respect to average scores on the factor about confidence in psychological help, people who rated their health as poor, and those who had an activity limitation, had higher scores compared to those who perceived their health as good, and those who had no activity limitation, respectively. People at risk of isolation had a lower average score on beliefs about the need of not coping alone (Factor 2), compared to those who reported more social support. People with lower levels of education had a lower score on predisposition to seek for psychological help (Factor 3), compared to those who had more years of education. The sensitivity analysis considering a cutoff of 6 or more points in the PHQ-9 for depressive symptoms, showed no difference in mean scores of the scale and each factor (total score, *p*-value = 0.4313; confidence, *p*-value = 0.3356; not coping alone, *p*-value = 0.0691; predisposition, *p-*value = 0.2185).

**Table 2:** Comparison of the ATSPPH-SF and factors mean scores, by characteristics of the sample

Variable	Total score		Confidence		Not coping alone		Predisposition	
Gender	Mean (SD)	*p*-value	Mean (SD)	*p*-value	Mean (SD)	*p*-value	Mean (SD)	*p*-value
Male	19.03 (4.22)	0.5621	7.09 (2.27)	0.5570	4.11 (1.67)	0.5120	7.82 (1.45)	0.9928
Female	19.37 (3.88)		7.28 (2.04)		4.27 (1.58)		7.82 (1.48)	
Age								
< 75	19.59 (3.67)	0.1463	7.39 (1.99)	0.1560	4.39 (1.57)	0.0548	7.80 (1.49)	0.8424
75+	18.82 (4.36)		6.99 (2.25)		3.98 (1.63)		7.85 (1.45)	
Education								
< 8	17.84 (5.27)	0.0863	7.06 (2.12)	0.6911	3.72 (1.97)	0.0739	7.06 (2.06)	**0.0056**
8	19.55 (3.59)		7.43 (1.92)		4.09 (1.50)		8.04 (1.26)	
12+	19.50 (3.73)		7.19 (2.18)		4.39 (1.53)		7.91 (1.34)	
Marital status								
Married	19.52 (4.26)	0.2159	7.25 (2.27)	0.3805	4.35 (1.71)	0.1427	7.92 (1.49)	0.0599
Single	18.39 (4.04)		6.90 (2.31)		3.61 (1.89)		7.87 (1.26)	
Divorced	18.56 (3.92)		6.93 (1.93)		4.37 (1.39)		7.27 (1.67)	
Widowed	19.82 (3.62)		7.55 (1.84)		4.26 (1.36)		8.02 (1.34)	
Living alone								
Yes	18.51 (4.11)	0.0827	6.85 (2.20)	0.1050	4.07 (1.66)	0.3766	7.59 (1.46)	0.1635
No	19.55 (3.90)		7.36 (2.07)		4.28 (1.58)		7.90 (1.47)	
Economic difficulties								
Yes	19.44 (3.68)	0.6508	7.54 (1.85)	0.0983	4.09 (1.56)	0.3451	7.81 (1.52)	0.9103
No	19.19 (4.13)		7.06 (2.22)		4.30 (1.62)		7.83 (1.44)	
Self-rated health								
Good	19.01 (3.90)	0.2137	6.99 (2.21)	**0.0357**	4.06 (1.70)	0.0597	7.96 (1.36)	0.0906
Not good	19.66 (4.05)		7.57 (1.92)		4.46 (1.44)		7.63 (1.60)	
Activity limitation								
Yes	19.81 (3.66)	**0.0499**	7.54 (1.99)	**0.0338**	4.37 (1.52)	0.2044	7.91 (1.40)	0.3879
No	18.79 (4.19)		6.95 (2.18)		4.10 (1.67)		7.74 (1.54)	
Frequency of visits to primary care								
Monthly	19.63 (3.79)	0.0976	7.43 (2.01)	0.0715	4.25 (1.65)	0.8071	7.95 (1.44)	0.1047
< Monthly	18.75 (4.19)		6.92 (2.22)		4.20 (1.53)		7.63 (1.50)	
Knows about mental health program								
Yes	19.89 (4.00)	0.1859	7.31 (2.17)	0.7908	4.53 (1.74)	0.0992	8.05 (1.35)	0.1641
No	19.12 (3.91)		7.23 (2.07)		4.15 (1.52)		7.75 (1.51)	
Depressive symptoms								
Yes	20.17 (3.73)	0.1467	7.81 (1.75)	0.0754	4.42 (1.56)	0.4419	7.94 (1.49)	0.5943
No	19.12 (4.00)		7.13 (2.15)		4.19 (1.61)		7.80 (1.47)	
Social support								
Risk of isolation	18.80 (4.19)	0.0527	7.09 (2.20)	0.2830	4.02 (1.68)	**0.0424**	7.69 (1.54)	0.1407
No risk	19.81 (3.65)		7.39 (2.00)		4.45 (1.48)		7.97 (1.38)	

In multivariate analysis (Table 3), we observed that people who had completed primary education (*p* = 0.0279), and those who had secondary education or more (*p* = 0.0168), had more positive attitudes towards seeking psychological help, compared to those with a lower level of education. Also, those who were at risk of social isolation (*p* = 0.0275), had less favorable attitudes. The sensitivity analysis, considering the 10-item scale showed similar results (See Supplementary Table 2, Additional file 1). In that analysis, the association with frequency of visits to the primary care also reached statistical significance (*p* = 0.0249), with people who visited the primary care once a month or more, having more positive attitudes towards seeking psychological help. The analysis with a cutoff of 6 or more points in the PHQ-9 for depressive symptoms, found similar results, with no association between depressive symptoms and attitudes towards seeking psychological help (8 item scale, *p*-value = 0.172; 10 item scale, *p*-value = 0.114).

**Table 3:** Factors associated with the attitudes towards seeking psychological help among older adults enrolled in primary care in Chile, according to multivariate linear regression

Variable (reference)	ATSPPH-SF (8 items)	
	B (SE)	*p*-value
Gender (male)		
Female	−0.406 (0.651)	0.5333
Age (< 75)		
75+	−0.494 (0.555)	0.3744
Education (< 8 years)		
8	1.963 (0.887)	**0.0279**
12+	1.955 (0.812)	**0.0168**
Marital status (married)		
Single	−0.333 (0.943)	0.7241
Divorced	−0.376 (0.834)	0.6531
Widowed	0.894 (0.715)	0.2129
Living alone (no)		
Yes	−1.071 (0.719)	0.1376
Economic difficulties (no)		
Yes	0.102 (0.553)	0.8543
Self-rated health (good)		
Not good	0.633 (0.565)	0.2640
Functional limitation (no)		
Yes	0.801 (0.557)	0.1517
Frequency of visits to primary care (monthly)		
< Monthly	−1.014 (0.532)	0.0579
Knows about mental health program (yes)		
No	0.615 (0.571)	0.2828
Depressive symptoms (no)		
Yes	1.114 (0.776)	0.1523
Social support (no risk)		
Risk of isolation	−1.211 (0.546)	**0.0275**

## Discussion

This study was aimed at assessing the attitudes towards seeking psychological help among older adults who are primary care users in Chile, and to determine which factors were associated with these attitudes. We used the ATSPPH-SF, and according to our results (mean 21.84, for the 10-item scale), older adults in Chile have more favorable attitudes towards seeking psychological help, compared to samples from other countries and younger age groups, which ranged between 13.90 and 20.45 [69, 70]. Although it has been reported that health care users have higher scores on this scale [69], and that older adults are more likely to seek mental health care, compared to younger groups [26, 71], more recent studies have found that older age is associated with less favorable attitudes towards seeking psychological help in Germany and China [24, 51]. On the other hand, there is no comparable information available about these attitudes among younger age groups in Chile.

We observed differences in the distribution of scores in factor 1, which expressed confidence in psychologist and the usefulness of psychological help. People with more health problems, particularly a negative self-rated health and difficulties to carry out daily life activities, had higher mean scores. It is possible that this group is more frequently in contact with primary care teams for regular checkups, and they are more likely to be referred to mental health consultation within the primary care, in case of need. Hence, we could hypothesize that this group of primary care users who have more physical health problems, are more familiarized with the interventions that psychologists provide, and they could consider it an available resource in case of mental health need.

In our study, social support increased the likelihood of favorable attitudes towards seeking psychological help. Social support has been previously identified as an important enabler of positive mental health seeking attitudes among older adults [26, 72]. An Australian study found that older adults with depression or anxiety symptoms who had a social support network, were more likely to be encouraged to seek mental health care [73]. Social connectedness could increase the likelihood of being aware of mental health symptoms, being in contact with family members or friends who have had direct experience with mental health services, and naturalizing mental health care seeking. According to our results, people who were at risk of social isolation had less favorable attitudes towards seeking psychological help, and had a lower mean score on factor 2, which referred to the importance of not coping alone with mental health problems. In their case, the lack of social networks might extend to perceived lack of resources within their community, including mental health services.

We also found an association between level of education and the attitudes towards seeking psychological help, being more favorable among people with at least primary education, compared to those with less than primary education. People with less years of education had lower mean scores on factor 3 of the ATSPPH-SF, which referred to the predisposition to seek psychological help, in case of mental health problems. Among studies that have focused on older adults, similar findings were observed in studies in Mexico [27], and Germany [24]. However, level of education was not associated with the attitudes towards seeking mental health care in another study from Germany [26], and a study from Australia [71]. None of these studies employed the ATSPPH-SF. A possible explanation for the tendency we observed is that older adults with less years of education could also have lower mental health literacy, in terms of recognizing symptoms of psychological distress, treatment availability, and alternatives to seek care. According to previous research, lower mental health literacy is associated with mental health stigma among older adults, and both factors are barriers for mental health seeking attitudes [74]. Another study found that mental health literacy mediates the effect of education on mental health services utilization [75]. It is also important to explore if this group of people are more likely to have experienced difficulties in access or negative interactions with health and mental health providers in primary care. Previous research has reported that lower level of education was associated with a more negative evaluation of primary care in Brazil and Malaysia [76, 77].

International studies have reported that anxiety and depression in older adults are underdiagnosed [7, 8]. Studies carried out in Chile before the COVID-19 pandemic have found that less than half of older adults with positive screen for depression had received a diagnosis [30, 31]. During the COVID-19 pandemic, the proportion of older adults in Chile with a positive screen for depression and anxiety increased [44], but their access to mental health care decreased [43]. Hence, addressing a higher demand for mental health care among older adults in Chile, and improving the ability to detect and treat mental health problems in this age group, should be a priority. According to previous research, psychotherapy can be an effective strategy to treat common mental disorders in old age [15–17], even more effective compared to other age groups [18].

Our results provide information to improve the access to psychological help among older adults in primary care, which is the most used health service among older adults in Chile [32]. Also, it is the place of access to mental health care, and a greater proportion of mental health care is provided at that level [37]. It is important to consider that, according to our study, older adults who are primary care users in Chile tended to express favorable attitudes towards psychological help. However, people with lower education and those at risk of isolation should be particularly considered, since they expressed less favorable attitudes towards seeking psychological help. Longitudinal studies have shown that social support has a positive effect on depression among older adults [78], and that it is a mediator of mental health in this group [79]. This stresses the importance of developing interventions to enhance social connection among community dwelling older adults in Chile, and to improve mental health literacy. Strategies aimed at reducing the risk and consequences of social isolation among older adults should be strengthened. In Chile, several programs to promote social integration, intergenerational relationships, and use of community network of social and health services for older adults have been implemented as part of social policies [80]. Along with these initiatives, programs developed in primary care may have an impact on social support availability, and on the attitudes of older adults to seek for psychological help. If these programs are part of an integrated care approach, they could improve the process of monitoring and referral to mental health consultation. Although the current community-based program to prevent disability among older adults in primary care (Más AMA) is focused on physical and cognitive risk of dependency, it has shown a perceived positive impact on mental health of participants [81]. Hence, similar community-based interventions, with a focus on promoting social connectedness and mental health among older adults who are users of primary care, apart from direct mental health benefits for users, could contribute to identify and address mental health needs of older adults in primary care.

It is important to consider how prepared are primary care services in Chile to address the needs of older adults. Existing negative stereotypes about old age, and limited recognition of the heterogeneity of the older population are among the challenges faced by public policies and health systems [70]. Negative stereotypes towards older adults have been found among mental health providers [82], which could be more marked in the case of patients with a lower level of education. This should be explored in future research and considered in interventions aimed at improving the skills of primary care teams to address the needs of older adults.

To the best of our knowledge, we employed the ATSPPH-SF for the first time in Chile. According to our analyses, this instrument maintains its ability to measure several dimensions of the attitudes towards seeking psychological help, as reported by the original study and others [46, 47], such as confidence in mental health professionals, openness or predisposition to seek for psychological help, beliefs about coping alone with emotional problems. Therefore, this scale was useful to have a first approach of attitudes towards seeking psychological help among older adults in Chile. However, it should be considered the need of developing an assessing tool more pertinent to Spanish language, the cultural and social context, and the organization and functioning of mental health services in Chile. It has been previously discussed that double barreled items included in this scale might be difficult to understand [48, 52]. Also, the use of simple phrases, and the avoidance of reversed or negation items have been recommended in general for this kind of scales [83, 84]. A mixed method approach could be an appropriate strategy, as suggested by a study that developed a scale for measuring cultural beliefs about psychotherapy patients in Chile [85].

It is important to mention some limitations of this study. First, the sample is non-probabilistic, and it was selected through a combination of methods, including random sampling of primary care users, and convenience sampling among participants of community groups. Therefore, this sample is not representative of the heterogeneity of older people living in the community and enrolled in primary care in Chile. Most participants in this study were adherent to programs for common chronic conditions that are treated in primary care. Compared with a representative sample of primary care users, a higher proportion of this sample had more years of education, and a lower proportion had depressive symptoms [86]. Also, a high proportion of participants were in contact with the health center more than once a year. Additionally, people who participate in older adult community groups might be over-represented. It is possible that people who accepted to take part in the study, were more likely to seek for psychological help than the general population of older adults who are primary care users. This could partly explain the favorable attitudes observed in this study. Hence, these limitations affect the generalizability of our estimations. Another limitation is the reduced sample size, which affected the statistical power of the study. In spite of this, our results showed consistency with previous findings about the factors associated with the attitudes towards seeking psychological help. It is important to further study these attitudes among older adults in Chile, considering a representative sample, to compare other groups, such as rural or immigrant population. Finally, due to our cross-sectional design, it was not possible to explore causal pathways between the factors assessed in our study and the attitudes towards seeking psychological help among older adults in Chile.

## Conclusions

The attitudes towards seeking psychological help among older adults who are primary care users in Santiago, Chile, were favorable. This suggests that other factors should be investigated, to determine what are the main barriers to access mental health care among this group. Community-based strategies to improve mental health literacy and social support and connection among older adults, could have an impact on the access to mental health care among people who have a lower level of education or are at risk of social isolation. These results provide insights on barriers that older adults who are primary care users might face to access psychological help in Chile, and in other countries with similar cultural background, where most of the mental health care is provided in primary care centers, and psychologists have a central role in mental health care teams.

### Supplementary Information

**Supplementary Material 1.**

## Data Availability

The anonymized datasets used and/or analysed during the current study are available from the corresponding author on reasonable request.

## Abbreviations

ATSPPH-SF: Attitudes Toward Seeking Professional Psychological Help Scale-Short Form
EFA: Exploratory Factor Analysis
CFA: Confirmatory Factor Analysis
