# Supplementary material for: Attitudes towards seeking psychological help among community dwelling older adults enrolled in primary care in Chile
Source: BMC Geriatr. 2024 May 1;24:386. doi: 10.1186/s12877-024-04986-3 (PMC11064339; doi:10.1186/s12877-024-04986-3)
Supplement: Supplementary file 1 — Supplementary Material 1. [file 12877_2024_4986_MOESM1_ESM.docx]

Sensitivity analysis

Supplementary Table 1. Comparison of the ATSPPH-SF scores between 8-item and 10-item scale

| Variable | 8-item (M 19.28, SD 3.97) | | 10-item (M 21.84 (SD 4.67) | |
| --- | --- | --- | --- | --- |
| Gender | Mean (SD) | *p*-value | Mean (SD) | *p*-value |
| Male | 19.03 (4.22) | 0.5621 | 21.71 (5.24) | 0.8028 |
| Female | 19.37 (3.88) |  | 21.88 (4.46) |  |
| Age |  |  |  |  |
| <75 | 19.59 (3.67) | 0.1463 | 22.25 (4.23) | 0.0961 |
| 75+ | 18.82 (4.36) |  | 21.21 (5.23) |  |
| Education |  |  |  |  |
| <8 | 17.84 (5.27) | 0.0863 | 20.13 (5.87) | 0.0783 |
| 8 | 19.55 (3.59) |  | 21.95 (4.11) |  |
| 12+ | 19.50 (3.73) |  | 22.17 (4.53) |  |
| Marital status |  |  |  |  |
| Married | 19.52 (4.26) | 0.2159 | 22.24 (4.98) | 0.1017 |
| Single | 18.39 (4.04) |  | 20.68 (4.49) |  |
| Divorced | 18.56 (3.92) |  | 20.73 (4.71) |  |
| Widowed | 19.82 (3.62) |  | 22.48 (4.12) |  |
|  |  |  |  |  |
| Living alone |  |  |  |  |
| Yes | 18.51 (4.11) | 0.0827 | 22.19 (4.64) | **0.0474** |
| No | 19.55 (3.90) |  | 20.80 (4.65) |  |
| Economic difficulties |  |  |  |  |
| Yes | 19.44 (3.68) | 0.6508 | 21.93 (4.97) | 0.6770 |
| No | 19.19 (4.13) |  | 21.67 (4.10) |  |
| Self-rated health |  |  |  |  |
| Good | 19.01 (3.90) | 0.2137 | 21.63 (4.67) | 0.4276 |
| Not good | 19.66 (4.05) |  | 22.12 (4.67) |  |
| Activity limitation |  |  |  |  |
| Yes | 19.81 (3.66) | **0.0499** | 21.42 (4.97) | 0.1585 |
| No | 18.79 (4.19) |  | 22.29 (4.30) |  |
| Frequency of visits to primary care |  |  |  |  |
| Monthly | 19.63 (3.79) | 0.0976 | 22.32 (4.43) | 0.0508 |
| < Monthly | 18.75 (4.19) |  | 21.10 (4.94) |  |
| Knows about mental health program |  |  |  |  |
| Yes | 19.89 (4.00) | 0.1859 | 22.59 (4.80) | 0.1281 |
| No | 19.12 (3.91) |  | 21.55 (4.60) |  |
| Depressive symptoms |  |  |  |  |
| Yes | 20.17 (3.73) | 0.1467 | 21.69 (4.73) | 0.2633 |
| No | 19.12 (4.00) |  | 22.64 (4.31) |  |
| Social support |  |  |  |  |
| Risk of isolation | 18.80 (4.19) | 0.0527 | 22.61 (4.40) | **0.0152** |
| No risk of isolation | 19.81 (3.65) |  | 21.13 (4.81) |  |

Supplementary Table 2. Comparison of the factors associated with the attitudes towards seeking psychological help among older adults enrolled in primary care in Chile, according to multivariate linear regressions

| Variable (reference) | 10-item scale | | 8-item scale | |
| --- | --- | --- | --- | --- |
|  | B (SE) | *p*-value | B (SE) | *p*-value |
| Gender (male) |  |  |  |  |
| Female | -0.698 (0.759) | 0.3592 | -0.406 (0.651) | 0.5333 |
| Age (<75) |  |  |  |  |
| 75+ | -0.763 (0.647) | 0.2394 | -0.494 (0.555) | 0.3744 |
| Education (<8 years) |  |  |  |  |
| 8 | 2.128 (1.034) | **0.0409** | 1.963 (0.887) | **0.0279** |
| 12+ | 2.294 (0.947) | **0.0162** | 1.955 (0.812) | **0.0168** |
| Marital status (married) |  |  |  |  |
| Single | -0.352 (1.100) | 0.7496 | -0.333 (0.943) | 0.7241 |
| Divorced | -0.637 (0.974) | 0.5133 | -0.376 (0.834) | 0.6531 |
| Widowed | 1.101 (0.835) | 0.1886 | 0.894 (0.715) | 0.2129 |
| Living alone (no) |  |  |  |  |
| Yes | -1.376 (0.839) | 0.1022 | -1.071 (0.719) | 0.1376 |
| Economic difficulties (no) |  |  |  |  |
| Yes | -0.336 (0.645) | 0.6032 | 0.102 (0.553) | 0.8543 |
| Self-rated health (good) |  |  |  |  |
| Not good | 0.589 (0.659) | 0.3729 | 0.633 (0.565) | 0.2640 |
| Functional limitation (no) |  |  |  |  |
| Yes | 0.782 (0.650) | 0.2301 | 0.801 (0.557) | 0.1517 |
| Frequency of visits to primary care (monthly) |  |  |  |  |
| < Monthly | -1.401 (0.621) | **0.0249** | -1.014 (0.532) | 0.0579 |
| Knows about mental health program (yes) |  |  |  |  |
| No | 0.822 (0.666) | 0.2187 | 0.615 (0.571) | 0.2828 |
| Depressive symptoms (no) |  |  |  |  |
| Yes | 1.308 (0.905) | 0.1496 | 1.114 (0.776) | 0.1523 |
| Social support (no risk) |  |  |  |  |
| Risk of isolation | -1.640 (0.637) | **0.0107** | -1.211 (0.546) | **0.0275** |
